# Mitogenomic organization and characteristics of deep-sea pelagic ostracods from both polar regions

**DOI:** 10.1186/s12864-026-12610-4

**Published:** 2026-02-27

**Authors:** Emily Yi-Shyuan Chen, Artur Burzyński, Beata Śmietanka, Marek Lubośny, Katarzyna Błachowiak-Samołyk

**Affiliations:** Institute of Oceanology, Polish Academy of Sciences, Sopot, 81-712 Poland

**Keywords:** Genetic diversity, Intron, Mitogenome, MtDNA, Ostracods, Pelagic zooplankton, Polar waters

## Abstract

**Background:**

Mitochondrial genomes are important markers that enable the study of evolutionary history, population structure, and biogeographic patterns in polar species, offering a framework to understand how polar organisms persist and adapt to extreme environments. However, they remain understudied in ostracods, an ecologically significant group of crustaceans. This study aimed to fill this gap by assembling and annotating the complete mitochondrial genomes of five pelagic ostracod species (*Boroecia maxima*, *B. antipoda*, *B. borealis*, *Discoconchoecia elegans*, and *Obtusoecia obtusata*) from the Arctic and Antarctic. Next-generation sequencing was applied, followed by detailed bioinformatic analyses to characterize gene content, structural features, and evolutionary patterns within these species.

**Results:**

The findings revealed a consistent gene arrangement across the five species, with the typical set of 37 genes, though some tRNA secondary structures showed species-specific variations. The mitogenomes displayed a high A + T content (71.9%–75.8%), had consistent Ka/Ks ratios under 1 indicating purifying selection, and included the unique presence of an intron in the *nd3* gene, a potential marker for the halocyprid family lineage. Haplotype diversity ranged from 0.791 ± 0.041 to 1.000 ± 0.034, with singleton haplotypes dominating in all species.

**Conclusions:**

These results provided new insights into the mitochondrial architecture of pelagic ostracods from polar waters. Overall, these genomic data lay the foundation for future comparative work on the population genetics and biogeography of these pelagic ostracods, particularly regarding the bipolar connectivity of this taxon.

**Supplementary Information:**

The online version contains supplementary material available at 10.1186/s12864-026-12610-4.

## Background

Polar marine ecosystems are characterized by extreme environmental conditions such as low temperatures, pronounced seasonality, and variable ice coverage [1, 2]. These factors shape biodiversity patterns and drive the evolution of distinctive traits in polar taxa, enabling survival and reproduction under extreme environmental conditions [2–4]. However, the genetic mechanisms underlying these traits remain largely unknown, highlighting the need to study polar organisms to gain insight into evolutionary processes in these unique ecosystems. Genomic research, of both nuclear and organellar genomes, provides important insights into how these organisms have evolved to cope with such extreme conditions and help understand their potential to respond to changing temperature regimes. Such studies range from showing that Arctic *Calanus* copepods have a larger body size, and therefore larger genome size, than their temperate counterparts [5] to providing evidence that positive selection events in polar species such as polar cod may shape physiological pathways such as stress responses to enhance survival in high-latitude systems [6–8]. Understanding how these cold-adapted and often non-model taxa persist in such environments requires further exploration of their underlying genetic architecture.

Maternal-inherited mitochondrial genomes (mitogenomes) are particularly useful for this purpose, as mitochondria are central to cellular energy metabolism and thermal regulation [6, 8]. Variations in mitochondrial genes can reflect evolutionary responses to environmental temperature and resource availability, serving as tools for investigating adaptation in polar ecosystems [5, 7, 8]. In metazoans, mitogenomes are typically comprised of 13 protein-coding genes (PCGs), two ribosomal RNA (rRNA) genes, and 22 transfer RNA (tRNA) genes, the general structure of metazoan mitochondrial genomes (i.e., mitogenomes) exhibit conservative gene content, minimal duplication, and a relatively rapid substitution rate [9, 10]. Mitogenomic analyses also establish consistent DNA-based molecular identification that helps resolve taxonomic ambiguities; this approach has already revealed cryptic diversity in various taxa from fish [11, 12] to smaller invertebrates like molluscs [13, 14] and tunicates [15]. Despite differences in ecology and physiology across taxa, the structure of the mitogenome is generally conserved and most metazoans show similar gene arrangements. There are taxa that display atypical structures, but the underlying causes do not always correlate to functional adaptation and instead reflect the deep evolutionary history of those groups [16, 17]. For example, some terrestrial arthropods are well-documented to have various tRNAs that deviate from the canonical cloverleaf structure, often missing the dihydrouridine arm, but remain functional [18]. Therefore, to accurately interpret if structural modifications are just a characteristic of a taxonomic group or if they represent an adaptation to streamline mitochondrial translation and cellular energy production for metabolic efficiency, there needs to be baseline genomic information for a broad range of taxa. These genomic data are often nonexistent for smaller and less conspicuous marine planktonic groups such as ostracods.

Marine ostracods are small (~ 1–5 mm), bivalved crustaceans that are globally distributed and ecologically important. With roughly 200 pelagic species formally described [19] and more than 2,000 extant species, they are a model group for environmental reconstruction and evolutionary studies due to their calcitic carapaces that preserve well in sediments, resulting in the most extensive fossil record for arthropods that date back to the Ordovician [20, 21]. There is even evidence that the benthic marine ostracod *Vargula hilgendorfii* can be a key reference organism for large-scale biogeographic studies [22]. Contrary to the focus on fossilized and benthic ostracods, morphological, ecological, and genetic data for living pelagic marine populations are critically lacking. In fact, the study by Nigro et al. [23] represents the most comprehensive analysis of the mitochondrial cytochrome oxidase subunit I (*cox1*) gene for pelagic ostracods, which assessed relationships within the group using 78 species. It demonstrated the benefit of DNA barcodes in species identification, given the difficulty of distinguishing based solely on morphological characters. Pelagic marine ostracods are highly sensitive to changes in temperature, salinity, and oxygen, making them good bioindicators with distinct distributions along environmental gradients in the water column [23–25]. Therefore, the next logical step to advance understanding of pelagic ostracods would be to fill in the gaps of genomic data to help determine the selective pressures driving their dispersal and distribution, particularly across polar and deep-sea habitats.

Yet despite their clear ecological and evolutionary significance, genomic resources for pelagic ostracods remain extremely limited. To date, there are 18 verified, complete mitogenomes representing seven species of the class Ostracoda publicly available in the National Center for Biotechnology Information (NCBI) GenBank repository [26]. Of these seven species, there are only four species from marine waters, with one unpublished mitogenome available for the order Halocyprida. This is a diverse order that includes Halocyprididae, the family of planktonic marine ostracods present in all the world’s oceans, ranging from the Arctic to the Antarctic and from surface waters to 9,307 m deep [20, 24], measuring only a few millimeters in length. Their abundance in marine zooplankton samples, often second only to copepods, means that effort is needed to compile molecular data for this ecologically important taxon. The current unavailability of marine ostracod sequences in public data repositories may be due to challenges in morphological identification, which is exacerbated when sampling in understudied polar environments, leading to taxonomic uncertainty and exclusion in routine plankton analyses [23, 27]. While there are ethanol-preserved ostracods present in museum collections, most were deposited decades ago initially in formalin, making the recovery of genetic information difficult and unreliable [28]. The four marine ostracod mitogenomes currently available in NCBI GenBank (i.e., *Vargula hilgendorfii*, *V. tsujii*, *Cypridina dentata*,* & Paraconchoecia oblonga*) are from temperate regions. Given the differences between temperate and polar marine habitats such as temperature adaptation, metabolic constraints, and evolutionary pressures, polar ostracods may exhibit distinct mitochondrial features that are not currently represented in existing GenBank Ostracoda data.

In this study, the complete mitochondrial genome sequences of five dominant halocyprid species in polar waters (i.e., *Boroecia maxima*, *B. borealis*, *B. antipoda*, *Discoconchoecia elegans*, and *Obtusoecia obtusata*) were assembled and annotated. The only non-exclusively polar species is *D. elegans*, which is a cosmopolitan species, but has established populations consistently found in polar waters. Because *D. elegans* individuals were solely taken from the high-Arctic, for the purposes of this study, it is therefore referred to as a polar species in this context. The objectives were to characterize the mitogenomic features of these ostracods collected from polar regions and to preliminarily identify any structural differences common amongst this planktonic group. By examining structural variation across multiple polar species within the same family, it will offer initial insights into divergence and highlight candidate mitochondrial features that may be conserved or divergent within polar lineages. This study provides baseline genomic data from a region that remains challenging to sample, setting important groundwork for subsequent population-level analyses on mitochondrial diversity and broader comparisons across polar taxa. In addition, to take advantage of the samples available, there was an effort to barcode the *cox1* gene, assess haplotype diversity and identify potential divergent lineages within the studied taxa. This also provided molecular confirmation of the morphological species identifications. The barcoding approach allows for a more comprehensive understanding of intraspecific genetic variation, which is essential for improving species delineation, detecting cryptic diversity, and refining reference databases for metabarcoding applications in routine zooplankton analyses.

## Methods

### Sample collection and DNA extraction

**Fig. 1:**
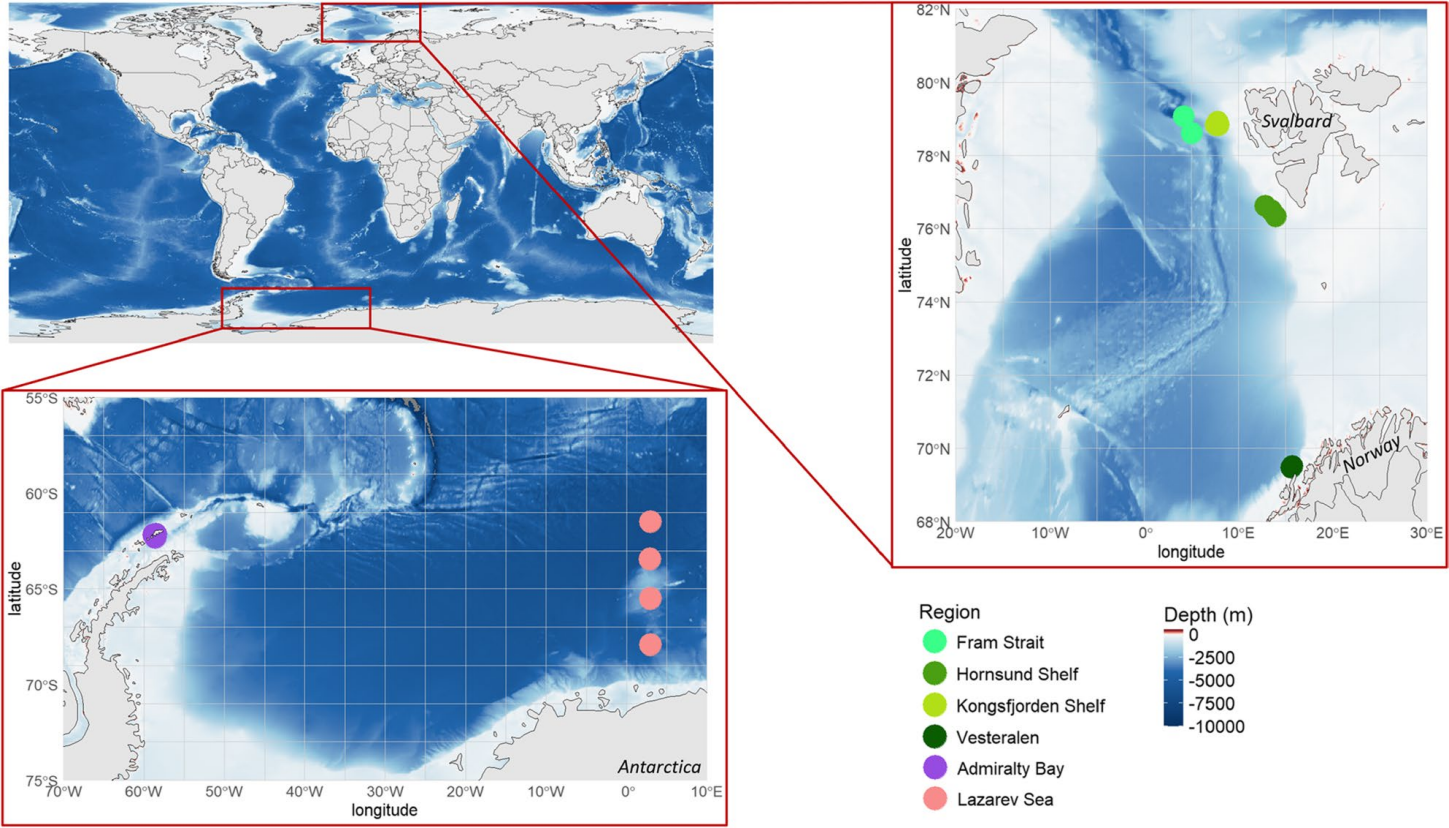
Locations of the 17 zooplankton sampling stations in the Arctic and Antarctic. Bathymetry is the ETOPO2 from the NOAA National Geophysical Data Center (www.ngdc.noaa.gov). Station information including IDs, dates, sampling depths, gear used, and ostracod count can be found in [Additional File 1]

All adult female ostracods used in analyses were sorted from ethanol-preserved bulk zooplankton samples collected from 17 stations in 6 distinct polar regions (Fig. 1). The station details, sampling gear, and associated metadata can be found in [Additional File 1]. Arctic individuals of *Boroecia maxima*, *B. borealis*, *Discoconchoecia elegans*, and *Obtusoecia obtusata* were taken from samples off the west coast of Svalbard from stations in Kongsfjorden, the Fram Strait, and Hornsund. Antarctic individuals of *Boroecia antipoda* were taken from samples off the coast of King George Island in the Admiralty Bay and the Lazarev Sea. Each individual ostracod was measured, photographed, then morphologically identified under a Leica M125 stereomicroscope to confirm sex, species, and life stage. Species-level identification was based on detailed morphological characters including but not limited to carapace shape, size ranges, frontal organ, and glands, following established species descriptions and monographs (e.g [29]). , , the Atlas of Atlantic Ocean Planktonic Ostracods [19], and the Atlas of Southern Ocean Planktonic Ostracods [30]. Adult females of each species were sorted out and preserved in ethanol until DNA extraction.

DNA was extracted from individual ostracods using the Sherlock AX isolation kit (A&A Biotechnology, Gdańsk, Poland), following the precipitation-based protocol provided by the manufacturer for fresh tissue samples. The modified lysis buffer contained 31.25 mM of dithiothreitol. This served as a reducing agent to disrupt disulfide bonds in proteins, enhancing proteinase K digestion and protecting DNA from enzymatic degradation. After extraction, DNA samples were stored at -20 °C until later use. DNA concentration and integrity were measured with an Epoch microplate spectrophotometer (BioTek Instruments, Winooski, VT, USA) and gel electrophoresis, respectively. The individual isolates presenting the best quality and concentration were selected for deep sequencing, the remaining ones were used in barcoding PCR.

### High-throughput sequencing

For each species, a DNA library was prepared from a single individual and sent to Macrogen Europe (Amsterdam, The Netherlands) for Next Generation Sequencing (NGS). The libraries were prepared with the TrueSeq Nano DNA kit and sequencing was performed with the NovaSeq X platform (Illumina, San Diego, CA, USA) with an insert size of 350 bp, generating paired-end reads with a read length of 150 bp. Sequencing-related quality assessments was performed on the raw sequencing data of each sample based on FastQC v.0.12.1 [31]. Optimal *de novo* circular assembly of mitogenomes were obtained from raw DNA reads using NOVOPlasty4.3.5 [32]. The seed sequences used for assembly were *cox1* sequences of the same species deposited in NCBI GenBank. If there were no sequences publicly available, the *cox1* Sanger sequences from this study served as the seed sequence. Set at program default values, proteins were predicted with CRITICA v.105b [33], Wise v.2.4.1 [34], and GLIMMER v.3.02 [35], and transmembrane domains in protein-coding genes were predicted using TMHMM v.2.0 [36]. Read coverage was calculated by mapping with Minimap2 [37] and SAMtools [38]. Ribosomal genes (rRNAs and tRNAs) were identified with Infernal v.1.1.5 [39] and Arwen v.1.2.3 [40]. The folding temperature of tRNAs was intrinsic to the custom mitoconstrictor workflow (https://github.com/aburzynski/mitoconstrictor), set using a heuristic parameterization based on nucleotide composition, and were not adjusted on a per-species basis. The custom mitoconstrictor package was also used to synchronize the annotations and visualize the circular mitochondrial diagrams. The annotations were inspected in Geneious Prime v.2025.0 (Dotmatics) and adjusted if necessary to reflect accurate positions.

### Sanger sequencing

To supplement the mitogenomic data from NGS and fill in barcode gaps in GenBank and BOLD, Sanger Sequencing targeting a ~ 680 bp fragment of the *cox1* gene region was completed for 129 individuals representing the five polar halocyprid species. The modified forward primer for ostracods, Ost-COI-1535F (5’-GGDGCHTGAAGWGCWATGYTAGG-3’) [23], in conjunction with the conserved universal reverse primer, HCO-2198R (5’-TAAACTTCAGGGTGACCAAAAAATCA-3’) [41] was used. PCR was performed in an Applied Biosystems VeritiPro Thermal Cycler in 50 µL volume of TaqNova KCl Reaction Buffer, containing 4 mM MgCl2, 0.2 µM of each primer, 0.2 mM of each dNTP, approximately 10–50 ng of template DNA, and 5 U of TaqNova polymerase. The PCR conditions were an initial denaturation at 95 °C for 3 min, 35 cycles of 94 °C for 45 s, 52 °C for 30 s, and 72 °C for 45 s, with a final extension of 72 °C for 5 min. PCR products were electrophoresed in 1% agarose gel and visualized after staining with ethidium bromide, purified using the ExoSAP-IT clean-up protocol (Thermo Fisher Scientific), and sent to Macrogen Europe (Amsterdam, The Netherlands) for Sanger Sequencing. An ABI 3730 automatic sequencer was used to resolve reaction products. The sequences were then BLASTed against the GenBank database to validate their identity [26, 42].

### Targeted *nd3* amplification and transcript verification

Specific primers (F_ND3ostra: 5′-AATCCTTTTGAGTGTGGT-3′; R_ND3ostra: 5′-TAATGAACCTTGTTGyCAC-3′) were designed for the *nd3* region based on assembled *Boroecia* mitogenomes. PCR on previously extracted genomic DNA was carried out using PCR Kit 5 (A&A Biotechnology) in 50 µL volume of PCR buffer I, containing 0.2 mM of each dNTP, 0.2 µM of each primer, approximately 10–50 ng of template DNA, and 2.5 U of RUN polymerase. The thermal cycling conditions were 95 °C for 1 min, followed by 40 cycles of 94 °C for 30 s, 52 °C for 30 s, and 72 °C for 30 s, with a final extension at 72 °C for 1 min. PCR products were purified using the PCR/DNA Clean-Up Purification Kit (EurX) and sent to Genomed S.A. (Warsaw, Poland) for Sanger sequencing.

For transcript analysis, total RNA was extracted from Ostracoda individuals using the GenElute™ Mammalian Total RNA Miniprep Kit (Sigma-Aldrich). Specimens were homogenized in 400 µL of Lysis Buffer containing ~1 mg/mL Proteinase K using a Bead Ruptor Elite (OMNI International) for 1 min at 4 m/s, incubated at 56 °C for 15 min, and processed following the manufacturer’s protocol. RT-PCR was performed using the SG OneStep RT-PCR Kit (EurX) in 50 µL volume of RT-qPCR SG Buffer, containing 50–100 ng RNA, 0.2 µM of each primer, and SG Enzyme Mix according to the manufacturer’s instructions. The 2× buffer provided with the kit included dNTPs. Reverse transcription was carried out at 50 °C for 20 min, followed by polymerase activation at 95 °C for 15 min, 40 amplification cycles (94 °C for 30 s, 52 °C for 30 s, 72 °C for 30 s), and a final extension at 72 °C for 1 min. Because PCR produced two bands (shorter from RNA transcript and longer probably caused by insufficient DNase I treatment) RT-PCR products were purified using the Agarose-Out DNA Purification Kit (EurX) and also sent to Genomed for sequencing.

### Bioinformatics and statistical analyses

Publicly available ostracod mitogenomes that were integrated for comparative analyses are as follows: *Vargula hilgendorfii* (AB114300) [43], *Cypridina dentata* (MK482395) [44], *V. tsujii* [45], *F. kushiroensis* (AP014656) [46], and *Heterocyprix spadix* (LC626010) [47]. *Cypridopsis vidua* (KP063117) and *Paraconchoecia oblonga* (PV231932) are unpublished.

To examine phylogenomic relationships, the 13 PCGs were aligned using MUSCLE [48] in MEGA11 [49]. Alignments were inspected and manually adjusted to maintain reading frame integrity. Phylogenetic trees were then constructed using Maximum Likelihood (ML) and Bayesian Inference (BI) approaches. The ML tree was constructed using IQ-TREE v2.2.6 [50] with 100,000 ultrafast bootstrap replicates and automatic detection of optimal thread usage based on a partitioned alignment. Best-fit model selection was performed automatically by ModelFinder for each partition. Mitogenome sequences from two outgroup species in the class Ichthyostraca, which belong to the same superclass as Ostracoda, were included to root the tree. For BI analysis, BEAST v.2.7.7 [51] was used. Input files were generated in BEAUti from partitioned alignments by gene, with a single Relaxed Clock Log Normal model applied to all loci, default MCMC settings, and a 10% burn-in. Various substitution models were tested, and all resulted in the same tree topology, indicating that model selection did not affect the inferred relationships. The final BI tree with posterior probability values was summarized in TreeAnnotator and visualized using FigTree v.1.4.5 [52]. The resulting ML and BI trees were presented as mirrored topologies.

For codon usage analysis, the Relative Synonymous Codon Usage (RSCU) was calculated using the ‘cubar’ package [53] in R (version 4.3.1) [54]. The annotated coding sequences from the mitogenomes were used as the input and RSCU values were then computed for each codon to assess codon usage bias across all PCGs. Post-annotated tRNA secondary structures were visualized using RiboSketch v.0.8.2 [55], and the ΔG values were calculated from the ViennaRNA package integrated within the custom mitoconstrictor package. Only tRNAs that showed consistent structural deviations (e.g., T-arm or DHU-arm loss) in four of the five species were produced into a figure.

To test for selective pressure acting on the mitogenome, all 13 PCGs were first aligned by MUSCLE in MEGA11 at the codon level. Alignments were inspected manually and trimmed to remove ambiguously aligned regions and gap-rich positions, retaining only positions corresponding to complete codons .For each PCG, the ratio of the number of nonsynonymous substitutions per nonsynonymous site (Ka) to the number of synonymous substitutions per synonymous site (Ks) was calculated. The Ka/Ks ratios were estimated with the ‘kaks’ function from the ‘seqinr’ package [56] in R (version 4.3.1) [54], which uses the method published by Li 1993 [57].

Sequences obtained from the Sanger sequencing were assembled using the Gap4 software from Staden Package v.1.7.0 [58] and aligned with ClustalX v.1.83 [59] in MEGA11. The aligned *cox1* sequences were trimmed to the same range. Intraspecific genetic diversity for the five species was estimated with the DnaSP v.6.12.03 software [60] using the *cox1* alignment. Standard diversity indices were calculated, including but not limited to number of haplotypes (H), polymorphic sites (S), and nucleotide diversity (π). The relationships between haplotypes were visualized with haplotype networks generated in the PopArt 1.7 program [61] using the Templeton, Crandall and Sing method [62].

## Results

### Mitogenome structure and gene arrangement

**Fig. 2:**
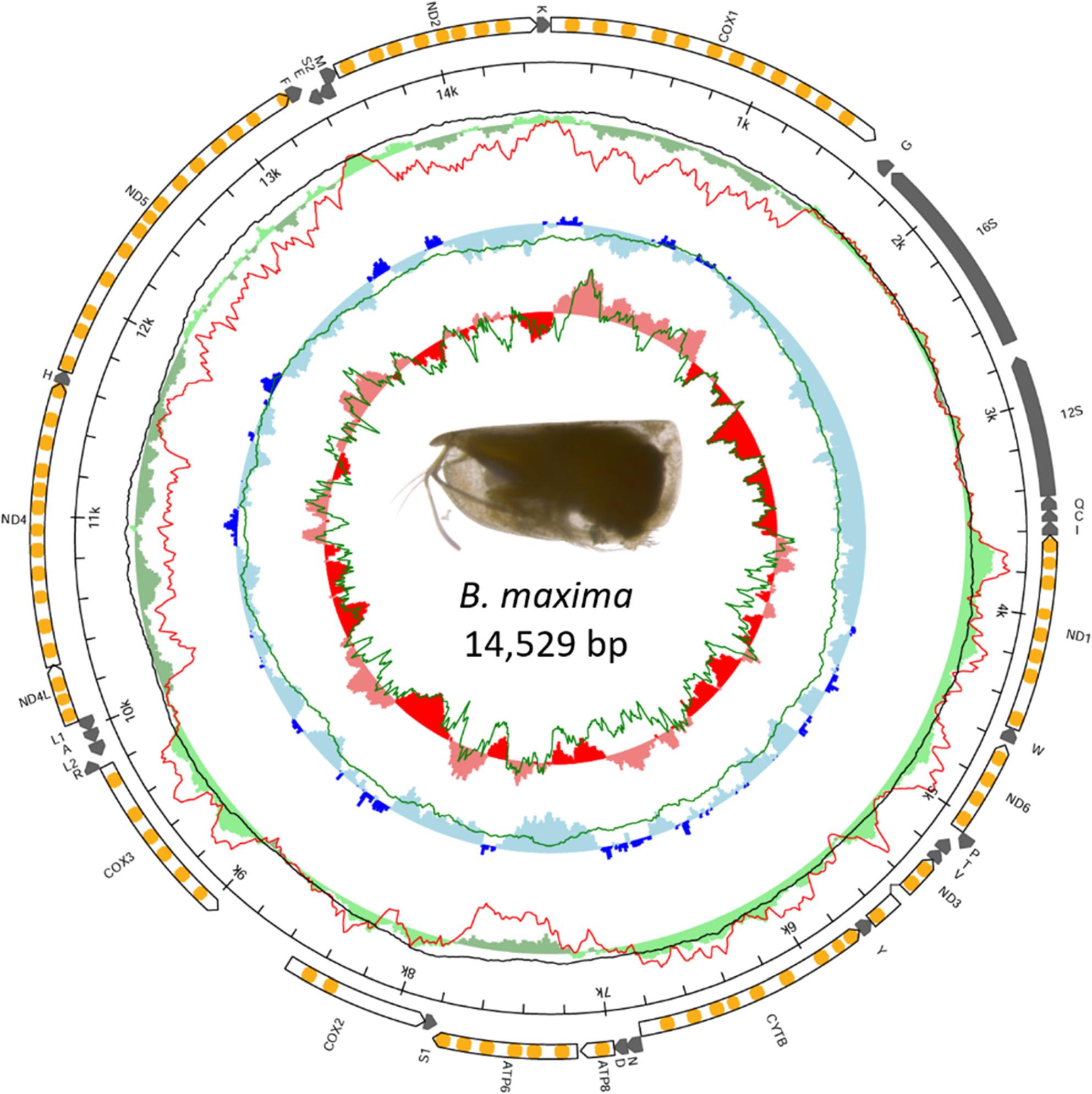
Mitogenomic map of *Boroecia maxima*, arbitrarily selected as a representative species for illustration purposes. Complete maps for each species are provided as full-page pictures in [Additional File 2: Supplementary Figs. 1 A-1E]. Within the white arrows representing PCGs, the predicted transmembrane domains of encoded proteins are marked in orange. The tRNAs and rRNAs are indicated by short and long gray arrows, respectively. The outer green circle illustrates the AT-skew; the filtered AT-skews are calculated at non-coding regions and the second codon position (red line) and at neutral and non-coding positions in a 1000 bp window (black line). The middle blue circle represents the GC-skew with the green line showing the skew calculated at neutral sites in a 1000 bp window. The inner red circle represents the local GC content with the green line showing the content at neutral sites

All species had the same gene content (Fig. 2) and the complete annotated mitogenomes can be found under the following accession numbers: *B. maxima* (PX686987), *B. antipoda* (PX686985), *B. borealis* (PX686984), *D. elegans* (PX686986), and *O. obtusata* (PX683731). The corresponding raw data deposited are available in the Sequence Read Archive (SRA) under the accession numbers SRX31118398 - SRX31118402 (BioSample SAMN53043039 - SAMN53043043). The three *Boroecia* species had the shortest lengths, ranging from 14,529 bp to 14,705 bp. All five species exhibited a strong but similar A + T bias, with AT contents ranging from the lowest 71.9% in *B. maxima* to the highest 75.8% in *O. obtusata*. The five mitogenomes had seven PCGs encoded on the heavy strand (*cox1*,* atp8*,* atp6*,* nd4l*,* nd4*,* nd5*, and *nd2*) and six on the light strand (*nd1*,* nd6*,* nd3*,* cytb*, *cox2*, and *cox3*).

The mitogenomic structure of the five polar species was similar, but the typical set of 13 PCGs and 22 tRNAs in metazoans was not initially identified with the annotation tools. Only 21 tRNAs out of 22 were detected in the three *Boroecia* species and *O. obtusata*, missing *trnQ*, while in *D. elegans*, the automatic annotations missed *trnV*, *trnR*, *trnL1*, *trnA*, and *trnL2*. These hypothetical secondary structures were manually derived from the mitogenome sequences according to the typical tRNA features. There were 12 PCGs detected in the three *Boroecia* species, missing *atp8*, which was manually identified based on sequence homology and then confirmed using BLASTX to match to known *stp8* sequences. An unusually high-coverage spike was observed between *cox3* and *nd4l in D. elegans*, coinciding with a localized GC content anomaly near a repetitive element [Additional File 2: Supplementary Figs. 1D, 4].

**Fig. 3:**
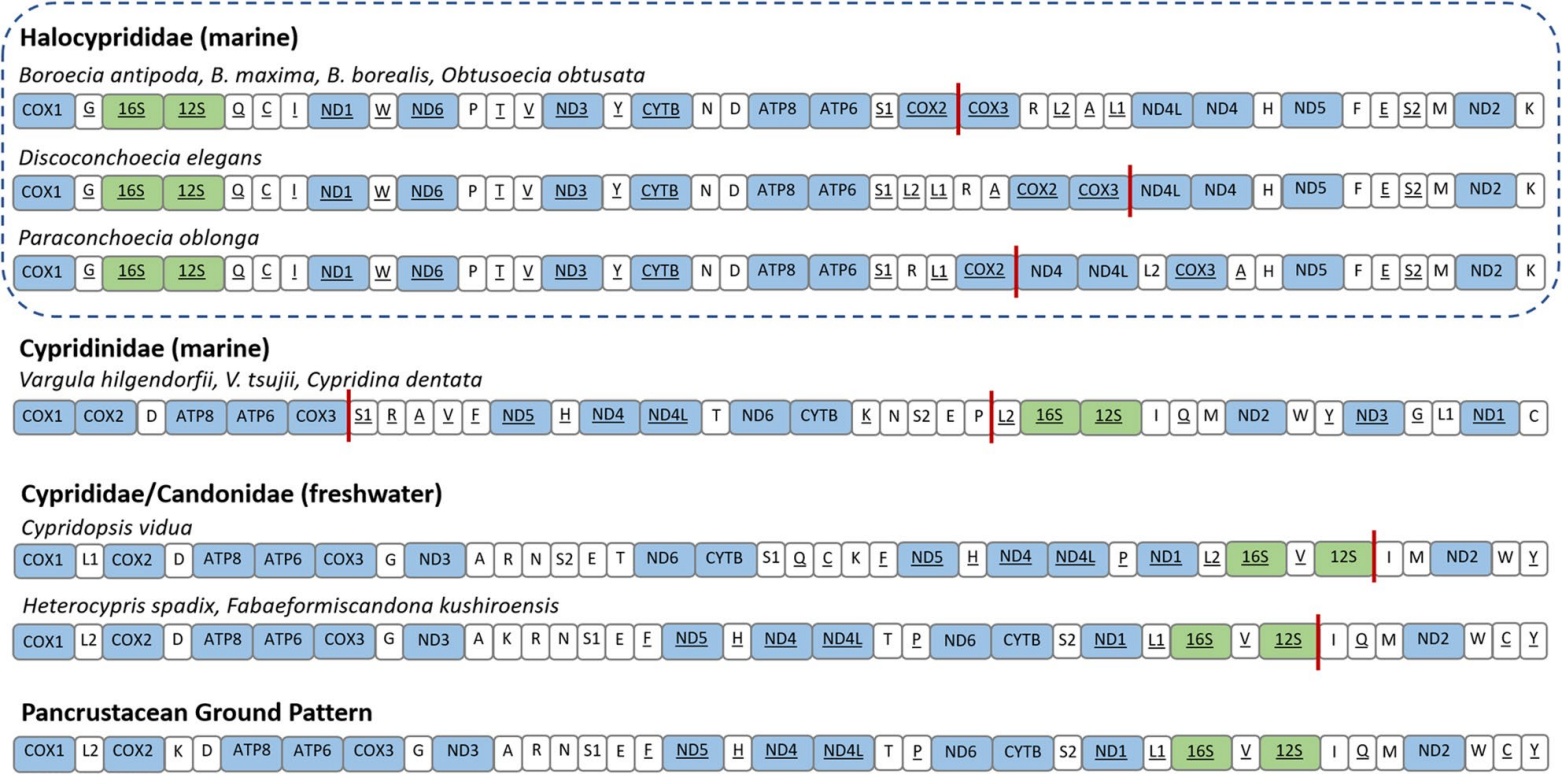
Qualitative gene order pattern comparison of the newly sequenced species of the family, Halocyprididae, with all other Ostracoda species that have publicly available, published mitogenomes in NCBI GenBank. The PCGs (blue), tRNAs (white), and rRNAs (green) are represented by color-coded boxes with genes encoded on the light strand underlined. The vertical red mark indicates location of non-coding regions. The pancrustacean ground pattern is based on the mitogenomes of *Homarus* and *Daphnia* [64]. For tRNA-Leu and tRNA-Ser genes, the anticodon-based naming convention is used (i.e., *trnS*(UCA) as *trnS1*, *trnS*(UCU) as *trnS2*, *trnL*(UAA) as *trnL1*, and *trnL*(UAG) as *trnL2*)

The five species in this study exhibited a PCG order that is similar but not identical to that of *P. oblonga*, in which *nd4* and *nd4L* were positioned between *cox2* and *cox3* (Fig. 3). The two rRNA genes, *rrnS* and *rrnL*, were sequential and positioned between tRNA genes in a conserved arrangement across the five mitogenomes, located between *trnG* and *trnQ.* A tabular output of the CREx analyses, run via the web-based platform Galaxy [63], showed the details of breakpoint identities and inferred rearrangement scenarios based on rearrangement types [Additional File 3: Sheet 3].

### *nd3* validation

The *nd3* gene in all five halocyprid ostracods contained a non-coding region consistent with an intron. Sanger sequencing of genomic DNA and reverse-transcribed RNA using the same primer pair showed the corresponding cDNA products were consistently shorter than genomic *nd3* amplicons, indicating removal of a non-coding sequence during RNA processing. Subsequent alignment revealed the intron boundaries, from nucleotide positions 155 to 217 within the *nd3* gene. The transcript lacked the internal region present in the genomic sequence, with the 5′ and 3′ exonic segments directly joined in the cDNA, forming a continuous open reading frame [Additional File 2: Supplementary Fig. 5, 6].

### Phylogeny

**Fig. 4:**
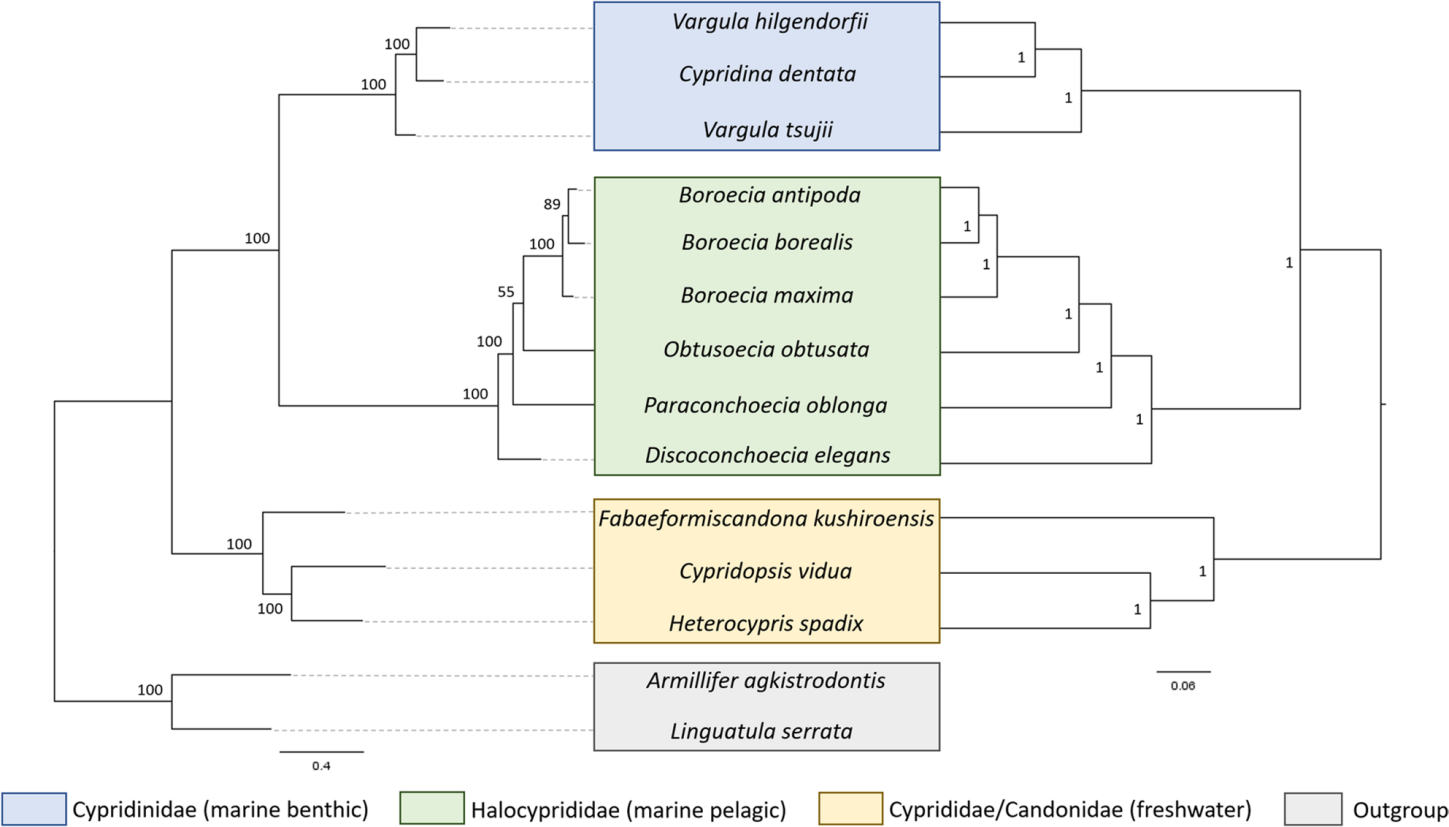
Phylogenomic tree constructed with ML (left) and BI (right) of the five species in this study together with all publicly available ostracod mitogenomes. The values for the ML and BI tree are the bootstrap values and the posterior probability values, respectively. For the ML tree, the scale represents the number of substitutions per site and for the BI tree, the scale reflects relative time and is not directly comparable to the ML scale. The color-coded legend corresponds to the different halocyprid families

The phylogenomic relationships constructed with both ML and BI methods produced mirrored topologies with high bootstrap and posterior probability values, respectively (Fig. 4). The benthic marine Cypridinidae, pelagic marine Halocyprididae, and freshwater Cyprididae and Candonidae each formed monophyletic, well-supported clades. The two outgroup sequences from species belonging to the superclass Ichthyostraca, were correctly placed outside Ostracoda, validating the rooting of the tree. Despite methodological differences in data partitioning, both ML and BI approaches recovered congruent topologies.

### Selection pressure, tRNA structure, and codon analyses

**Fig. 5:**
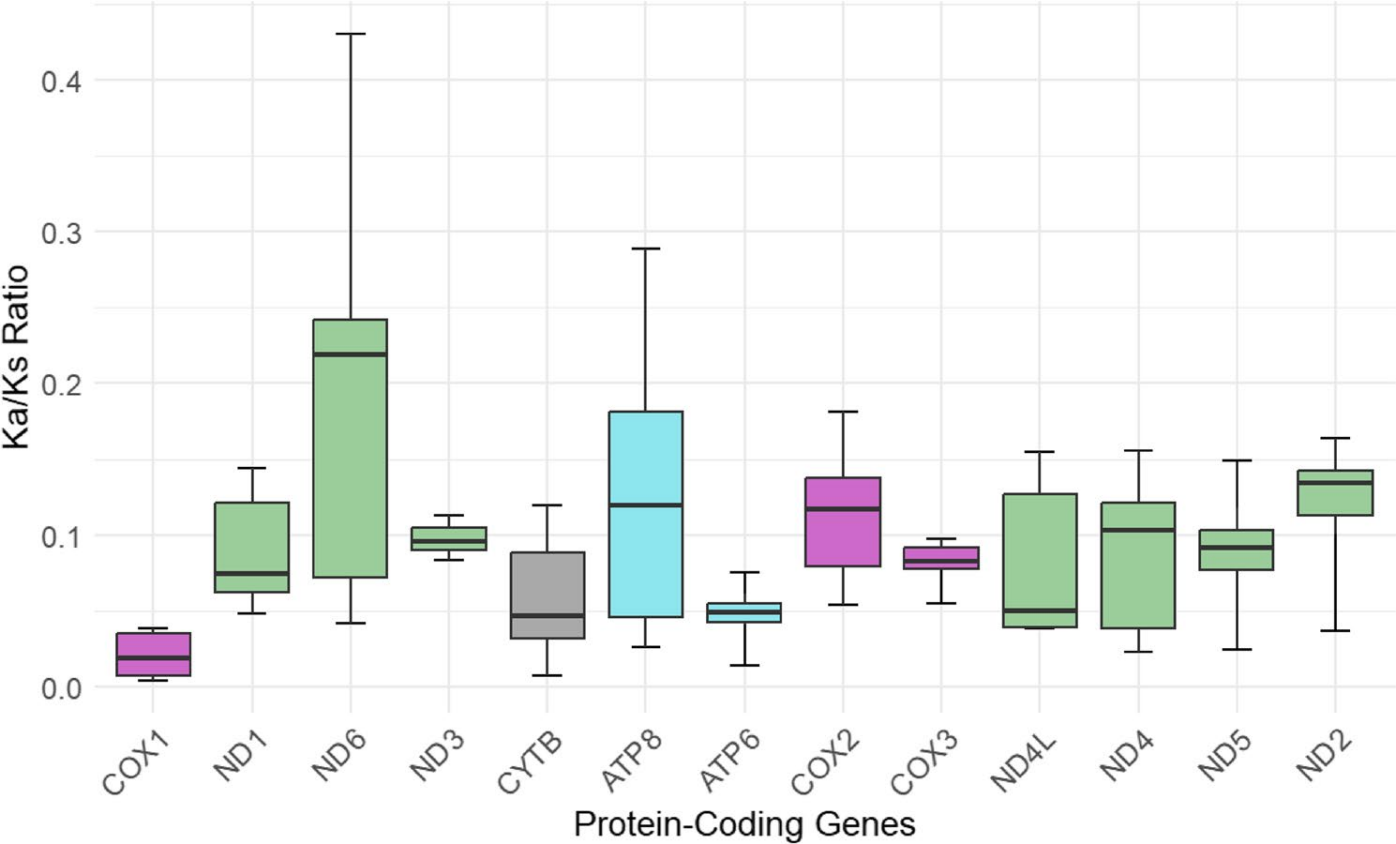
Ka/Ks ratios across the set of PCGs for the five halocyprid mitogenomes. Boxes are colored by OXPHOS complexes (Complex I: NADH dehydrogenase – green, Complex II: Cytochrome b – gray, Complex IV: Cytochrome C oxidase – magenta, Complex V: ATP synthase – cyan)

Selective pressure analyses on the mitogenomes showed that all PCGs had Ka/Ks ratios below 1, consistent with purifying selection (Fig. 5). Overall Ka/Ks values ranged from 0.0046 (*cox1*) to 0.4303 (*nd6).* The lowest ratios were of *cox1* (Ka/Ks < 0.05 across all samples) while *nd6* and *atp8* exhibited the highest mean Ka/Ks ratios of 0.185 and 0.113 respectively [Additional File 3: Sheet 2].

Of the 22 tRNAs, there were 10 whose secondary structures were consistently canonical in all species: tRNA-Gly, tRNA-Ile, tRNA-Leu1, tRNA-Leu2, tRNA-Met, tRNA-Asn, tRNA-Pro, tRNA-Gln, tRNA-Trp, and tRNA-Tyr. Another five tRNAs (i.e., tRNA-Cys, tRNA-Asp, tRNA-Phe, tRNA-His, and tRNA-Ser1) displayed the loss of the T-arm across all species. The remaining seven tRNAs that varied in aberrant structures between the species were visualized [Additional File 2: Supplementary Fig. 3]. For example, *B. borealis* was the only species with a canonical structure for tRNA-Ala while the other four species lacked the T-arm. Some of these seven tRNAs such as tRNA-Lys and tRNA-Val also exhibited highly reduced variable regions of 1–3 nucleotides despite an otherwise cloverleaf structure. Across all species, there were 7 pairs of U-U, 1 pair of A-A, and 2 pairs of G-U mismatches present in the seven tRNAs.

The RSCU analysis revealed strong codon usage bias across amino acids, generally favoring codons ending in T or A [Additional File 2: Supplementary Fig. 2]. Codons like TTT (Phe), TTA (Leu), and TCT (Ser) consistently showed high RSCU values (> 1.7). For example, for Leu, the codons TTA and CTA showed elevated RSCU values (up to 1.89 and 1.71 respectively), while CTC, CTG, and TTG were underrepresented (RSCU < 0.57) [Additional File 3: Sheet 1].

### Intraspecific diversity

**Fig. 6:**
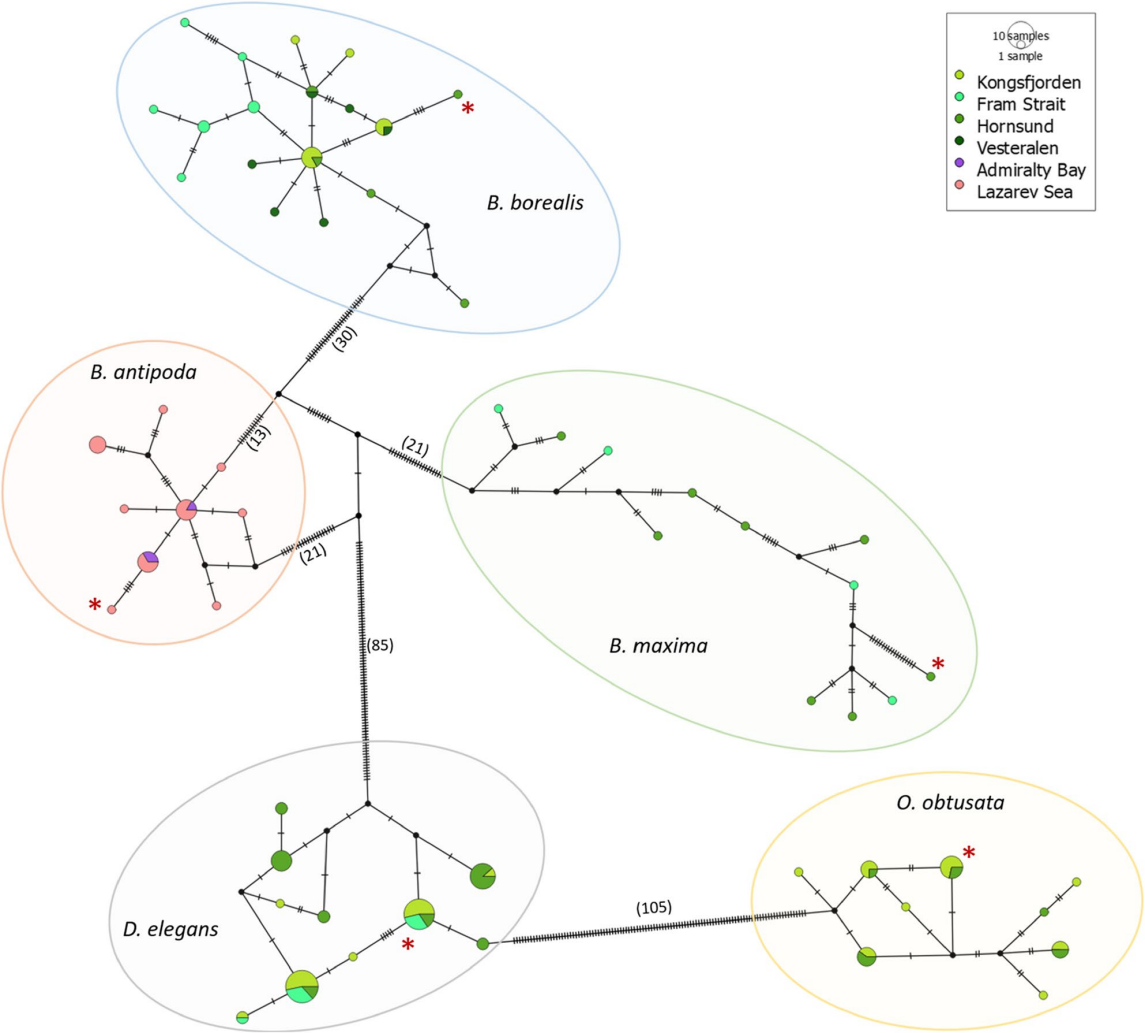
Combined haplotype network of the five halocyprid species based on *cox1* sequences. Each circle represents a unique haplotype, with circle size proportional to haplotype frequency. Hatch marks on connecting lines indicate the number of mutational steps between haplotypes; where distances are large, the number of steps is indicated in parentheses. Colored outlines group the clusters corresponding to individual species: *B. maxima* (green), *B. borealis* (blue), *B. antipoda* (pink), *O. obtusata* (yellow), and *D. elegans* (gray). Asterisks indicate individuals selected for NGS. A color code corresponding to sampling locations that follows the same color scheme as Fig. 1 is provided

A fragment of the *cox1* gene was successfully sequenced from 129 female individuals representing the five halocyprid species, with a length of 586 bp post-trimming. The *cox1* data generated in this study can be found in GenBank under the PX507539 - PX507666 accession numbers The mt-*cox1* haplotype networks consisted of a total of 58 haplotypes across the 129 samples, with the species-level breakdown seen in Table 1. Instead of a typical stellate shape with a main haplotype representing the majority of the network for each species, unique single haplotypes dominated for all species (Fig. 6). This was most pronounced in *B. maxima*, where all 12 samples were singletons. Haplotype diversity (Hd) ranged from 0.791 ± 0.041 in *D. elegans* to 1.000 ± 0.034 in *B. maxima*, indicating a high level of haplotype richness within the sampled populations. The high values for nucleotide diversity (π), and average number of nucleotide difference between *cox1* haplotypes (K) further supports the presence of substantial genetic variation in mitochondrial DNA. Tajima’s D and Fu’s Fs values ranged from negative to positive across species, suggesting variability in allele frequency distributions. None of the Tajima’s D or Fu’s Fs values were statistically significant (Tajima’s D and Fu’s Fs coalescent-based probabilities: *p* > 0.10) and indicated no strong deviation from neutrality.

**Table 1:** Genetic diversity indices estimated with DnaSP v.6.12.03 based on the *cox1* sequences obtained for the five halocyprid species analyzed in this study. The statistical significance for Tajima D and Fu’s Fs neutrality tests was assessed with a standard threshold of *p* < 0.10

	*B. antipoda*	*B. borealis*	*B. maxima*	*D. elegans*	*O. obtusata*
Samples (*N*)	22	29	12	41	25
Number of Haplotypes (H)	9	18	12	10	9
Polymorphic Sites (S)	21	25	44	13	14
Haplotype Diversity (Hd)	0.844 ± 0.048	0.941 ± 0.029	1.000 ± 0.034	0.791 ± 0.041	0.857 ± 0.039
Nucleotide Diversity (π)	0.0069 ± 0.0013	0.0068 ± 0.0009	0.0256 ± 0.0045	0.0071 ± 0.0004	0.0078 ± 0.0009
Average Number of Nucleotide Differences (K)	4.0433	3.9852	15.0000	4.1732	4.5467
Tajima’s *D*	-1.1131	-1.4309	-0.2580	1.1599	0.7854
Fu’s Fs	-0.458	-9.056	-3.545	0.552	0.299

## Discussion

### Mitogenome architecture and functional features

The overall nucleotide compositions of the five halocyprid mitogenomes from this study showed strong A-T biases, ranging from 71.9% to 75.8%. These values were substantially higher than those reported for marine ostracods from different families, which have A + T contents of 61.6% in *V. hilgendorfii* [43], 64.6% in *V. tsujii* [45], and 67.96% in *C. dentata* [44]. This suggests that halocyprid mitogenomes may be characterized by a generally higher A + T content relative to other ostracod lineages. Supporting this pattern, the only other halocyprid mitogenome publicly available, *P. oblonga* (accession no. PV231932), also displays a similarly high A + T content of 74.5%, falling well within the range of the newly sequenced species. These compositional differences in halocyprids are mirrored by unique gene arrangement within the mitogenome. The three *Boroecia* species displayed lengths all shorter than 14,800 bp, which is shorter than previously published ostracod mitogenomes in GenBank, which range from 15,205 − 16,783 bp. In contrast, the mitogenomes of *D. elegans* and *O. obtusata* fall within this previously known range at 16,050 bp and 15,494 bp, respectively.

The gene arrangement for Halocyprididae was substantially different from the ancestral pancrustacean model, with numerous positional changes and inversions, while the other ostracod families such as Candonidae and Cyprididae demonstrated closer alignment with the ground pattern. Gene arrangements in metazoan mitochondrial genomes are generally conserved, but many crustaceans are well-documented to exhibit notable variations in gene order [65–67] and duplicated control regions [22]. These deviations from the pancrustacean ground pattern likely do not impact protein function directly, but may indicate shifts in mitochondrial regulation or transcriptional processing and highlight the evolutionary plasticity of mitogenomes. The identical order of PCGs across the five polar halocyprid species indicates a conserved mitochondrial gene arrangement within this polar clade. In contrast, the only available non-polar halocyprid mitogenome, *P. oblonga*, differed in PCG order, including the repositioning of *cox2* and *cox3* and a translocation of the nd4 and nd4l gene block (Fig. 3). Because all species considered here are pelagic halocyprids, these differences are unlikely to reflect ecological habitat. Instead, the shared PCG order across all five polar species, despite their geographic separation between the Arctic and Antarctic, suggests conserved genomic organization associated with this high-latitude lineage, potentially maintained by functional properties [68] or phylogenetic constraints [69]. Future sequencing of additional non-polar halocyprid species will be critical to clarify whether this arrangement is specific to polar halocyprid species or represents a wider but unique pattern within this family.

An unusual feature seen in all halocyprids was the presence of a split *nd3* gene. Upon closer inspection, these splits were not a result from gaps in coverage nor assembly issues, but intron presence was independently confirmed by concordant genomic and cDNA sequencing, an approach rarely applied in mitochondrial genome studies of small, ethanol-preserved marine invertebrates. Mitochondrial introns are well known in early-diverging metazoans, particularly nonbilaterian phyla such as Cnidaria and Porifera, where genome architectures are more variable, often including fragmented genomes, RNA editing, and intronic sequences [70–73]. However, the confirmation of an intron within the *nd3* gene in five halocyprid ostracods represents a significant deviation from the typical bilaterian mitochondrial architecture, which is generally characterized by compact, intronless genomes [74]. A comparable case of an intron in a bivalve mitochondrial gene was previously documented [75], indicating that introns may be more widespread in bilaterians than previously appreciated, albeit still extremely rare. The discovery of the intron in the *nd3* gene of a bilaterian taxon, the Halocyprida (Ostracoda), supported this view and extended the relevance of their observations that unusual mitochondrial features may not limited to deep-branching animal lineages but also persist in overlooked bilaterian taxa that are under-sampled or morphologically cryptic. Regardless of the evolutionary origin, the conserved presence of this *nd3* intron across five halocyprid species suggests functional retention and that it may be a characteristic genomic trait within halocyprid ostracods. Interestingly, the GenBank record of the temperate halocyprid species *Paraconchoecia oblonga* (accession no. PV231932) may also contain a similar *nd3* gene split, although this feature has not been formally reported in the literature. Since the intron was effectively spliced and did not disrupt the open reading frame, this finding adds to a small but growing body of literature that challenges the long-standing notion of bilaterian mitochondrial simplicity. It will be important to continue exploration of mitochondrial genomes across the animal tree, including well-known lineages, and integrate transcriptomic validation into genome annotation.

### Phylogenomic relationships and selective pressures

The conserved gene order among all five polar species in this study became more compelling when viewed alongside their phylogenetic proximity. The inclusion of a phylogenomic analysis based on full mitochondrial genomes was essential for multiple reasons. It first served as a validation step, confirming the taxonomic identity and mitochondrial origin of the assembled genomes; the consistent grouping of the five polar species within marine pelagic halocyprids supported the accuracy of the assemblies and their annotations. The tree topology also provided a valuable assessment of the utility of mitochondrial data for resolving ostracod evolutionary relationships. Despite the limited species with genomic data available relative to overall diversity of ostracods, the clear separation of benthic, pelagic, and freshwater clades suggests that mitochondrial genomes retain sufficient phylogenetic signal at higher taxonomic and ecological scales. Moreover, congruence between the ML and BI topologies even when different partitioning and models were used added confidence to the robustness of these findings (Fig. 4).

The widespread purifying selection in the PCGs of the newly sequenced species was consistent with a pattern commonly found in other crustaceans [76–79]. The highest Ka/Ks ratios were in *atp8* and *nd6*, suggesting that these two genes experienced lower levels of selective pressure compared to the other PCGs, and *cox1* was indicated to be under the strictest selection constraints (Fig. 5). This was not unusual, as *atp8* and *nd6* have been reported to be the most rapidly evolving and *cox1* to be the slowest evolving PCG in marine species, from vertebrates [80, 81] to a wide range of invertebrates [78, 82, 83]. Overall, these patterns highlight gene-specific differences in evolutionary rates, which may inform future studies on mitochondrial markers for phylogenetics and species delimitation in halocyprid ostracods.

While there were some wobble and atypical base pairings observed in the predicted tRNA secondary structures during folding, this is not uncommon [67, 76, 84] and the tRNA can remain functional [16]. Of the set of 22 tRNAs, 10 displayed the canonical structure for all species, but the consistent absence of full cloverleaf structures in several tRNAs supports previous findings that many arthropods tolerate or even rely on reduced tRNA structures [16, 18, 85]. It cannot be determined whether these truncated forms result in specific energetic or translational advantages in cold environments without future empirical tests and validation, but their presence here across geographically distinct polar halocyprids could potentially be a stable inherited feature.

Another consistent feature in the five mitogenomes was the codon usage patterns. Despite minor fluctuations in values, the RSCU showed a similar distribution among the five species, with codons ending in A or T generally overrepresented. While an overrepresentation of codons ending in A or T is generally expected given the high AT content of mitochondrial genomes, the observed RSCU distributions suggest that codon usage bias may exceed what would be predicted by nucleotide composition alone. This relationship is also reflected in the inner skew plots of the mitogenome maps [Additional File 2: Supplementary Figs. 1 A-1E], which show consistent AT-skew across all species. Furthermore, the two most abundant codons for all species were Leu (TTA) and Phe (TTT), reinforcing presence of the AT bias typical of invertebrate mitochondrial genomes [77–79]. These trends likely reflect non-random codon usage patterns, potentially driven by underlying mutational pressures rather than selection for translational efficiency, since tRNA availability and genome structure constrain codon choice [86, 87]. In addition, codon-anticodon interactions may play a self-amplifying role in shaping codon usage, contributing to the observed bias beyond simple compositional constraints.

### Intraspecific diversity and broader implications

Although this dataset includes only one species from the Southern Ocean (*B. antipoda*) and two congeners from the Arctic (*B. maxima* and *B. borealis*), the *cox1* data reveal high haplotype richness and a lack of clearly dominant haplotypes across the five polar halocyprid species examined. While the limited sample sizes and uneven geographic coverage do not allow formal assessment of population-level structure or connectivity, the observed haplotype networks (Fig. 6) are characterized by numerous unique haplotypes separated by multiple mutational steps, including highly divergent singletons. Similar network shapes have been reported for other polar and deep-sea marine taxa, such as the Arctic amphipod *Themisto abyssorum* [88] and the Antarctic polychate *Harmothoe crosetensis* [89]. With 18 unique haplotypes out of 29 individuals in *B*,* borealis*, this aligns with *Porroecia spinirostris*, a temperate pelagic ostracod species, from a concentrated region in the South China Sea where 36 haplotypes from 85 individuals were observed [90]. These results contrast with [91], which found that the benthic ostracod *Polycope pseudoinornata* from the Arctic and sub-Arctic in the North-Atlantic exhibited low haplotype diversity and showed no clear geographic connection, despite a broader sampling area. The halocyprids sampled showed high haplotype diversity, consistent with expectations for pelagic taxa with high dispersal potential. Specimens were collected from six distinct polar locations, representing a broad spatial range. Wide dispersal via ocean currents may facilitate gene flow among populations, which can limit the accumulation of strong geographic genetic structure. However, the total of 12–41 individuals per species means did not permit robust inference of population connectivity or the influence of geographic distance and hydrography. Future studies that include larger sample sizes across these locations would allow for more detailed analyses on a population-scale. Nevertheless, this study provided a uniquely extensive collection of halocyprids at both poles from polar waters that are particularly challenging to sample consistently. This collection and results offer an important foundation for future research for direct assessments of bipolar connectivity to better understand ostracod evolution, biogeography, and ecology.

No cryptic species were detected in this study, but the combination of observed gene order variations relative to the ancestral pancrustacean model, high haplotype richness, and consistent structural variation in several tRNAs across species suggests potential mitogenomic variability within Halocyprididae. Together with the intron observed in *nd3*, these patterns may reflect both lineage-specific evolutionary pressures and the role of high-latitude environments in shaping mitochondrial genome organization. Halocyprids are only a couple millimeters in size, so polar systems present extreme physiological challenges, including chronically low temperatures, seasonal food and light variability, and high energetic demands for locomotion and reproduction [1, 3, 8]. Mitochondrial function is the key to maintaining energy balance under such conditions, and variation in mitogenome architecture could reflect selective pressures acting to optimize energy production and oxidative stress management. Although the current study does not directly link mitochondrial structural traits to cold adaptation, consideration of these selective pressures underscores the broader relevance of mitogenomic data for understanding how polar plankton adapt to energetically constrained ecosystems. Comparative studies with wider geographic ranges and the incorporation of nuclear data will be needed to resolve the extent of divergence and test hypotheses of bipolarity in these groups. However, given the frequent exclusion of marine pelagic ostracods from community analyses due to taxonomic uncertainty, particularly in polar samples, the genetic resources presented here in this study will improve species identification and ecological interpretation in future monitoring efforts. The generation of verified mitogenomes and *cox1* sequences from morphologically identified individuals also strengthens the reference databases needed for taxonomic assignment of environmental DNA and high-throughput sequencing datasets.

## Conclusion

This study presented the first complete mitochondrial genomes of polar planktonic ostracod species, filling a significant taxonomic gap in the representation of Ostracoda in polar marine genetic resources. All genomes were relatively similar in size and structure within the Halocyprididae family, but distinctly differentiated from other ostracod families. Transcript verification confirmed both the gene’s expression and the presence of a conserved intron in the *nd3* gene of this family, adding another case of intron retention in metazoan mitogenomes. The monophyly of Halocyprididae was supported by phylogenetic results and consistently clustered the five polar species, indicating their tight evolutionary connection. Together, these results advance our understanding of ostracod mitochondrial evolution and provide a foundation for deeper population genomic, phylogeographic, and metagenomic studies of lesser-studied zooplankton, especially in high-latitude systems.

## Supplementary Information

Supplementary Material 1.

Supplementary Material 2.

Supplementary Material 3.

## Data Availability

The annotated mitochondrial genome sequences can be found in GenBank with the following accession numbers: *B. maxima* (PX686987), *B. antipoda* (PX686985), *B. borealis* (PX686984) *, D. elegans* (PX686986) *,* and *O. obtusata* (PX683731) **.** The *cox1* data generated in this study can be found in GenBank under the PX507539 - PX507666 accession numbers. The raw data submitted to the SRA can be found in GenBank under the accession numbers SRX31118398 - SRX31118402 (BioSample SAMN53043039 - SAMN53043043), with all five files stored in the BioProject accession number PRJNA1355481.
