# Supplementary material for: Mitogenomic organization and characteristics of deep-sea pelagic ostracods from both polar regions
Source: BMC Genomics. 2026 Feb 27;27:368. doi: 10.1186/s12864-026-12610-4 (PMC13067483; doi:10.1186/s12864-026-12610-4)
Supplement: Supplementary file 2 — Supplementary Material 2. [file 12864_2026_12610_MOESM2_ESM.pdf]

Mitogenomic organization and characteristics of deep-sea pelagic ostracods from both polar regions

Emily Yi-Shyuan Chen*, Artur Burzyński, Beata Śmietanka, Marek Lubośny, and Katarzyna Błachowiak-Samołyk

1A.

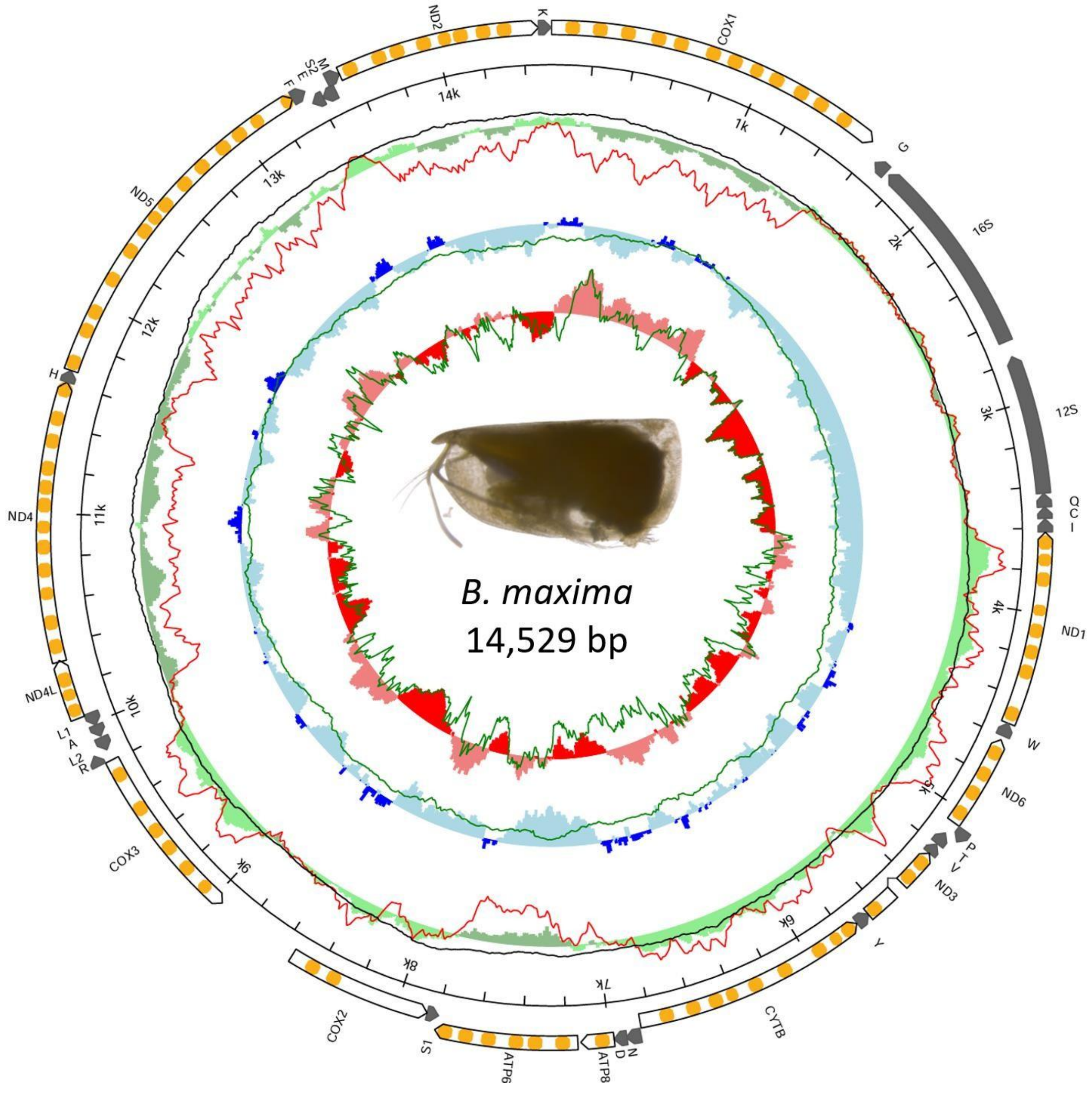

Supplementary Figures 1A—1E: Mitogenomic maps of the five species from this study presented as full-page images. The process and tools used for generating the figures can be found in the Methods section. 1A. *Boroecia maxima*, 1B. *B. borealis*, 1C. *B. antipoda*, 1D. *Discoconchoecia elegans*, 1E. *Obtusoecia obtusata*

1B.

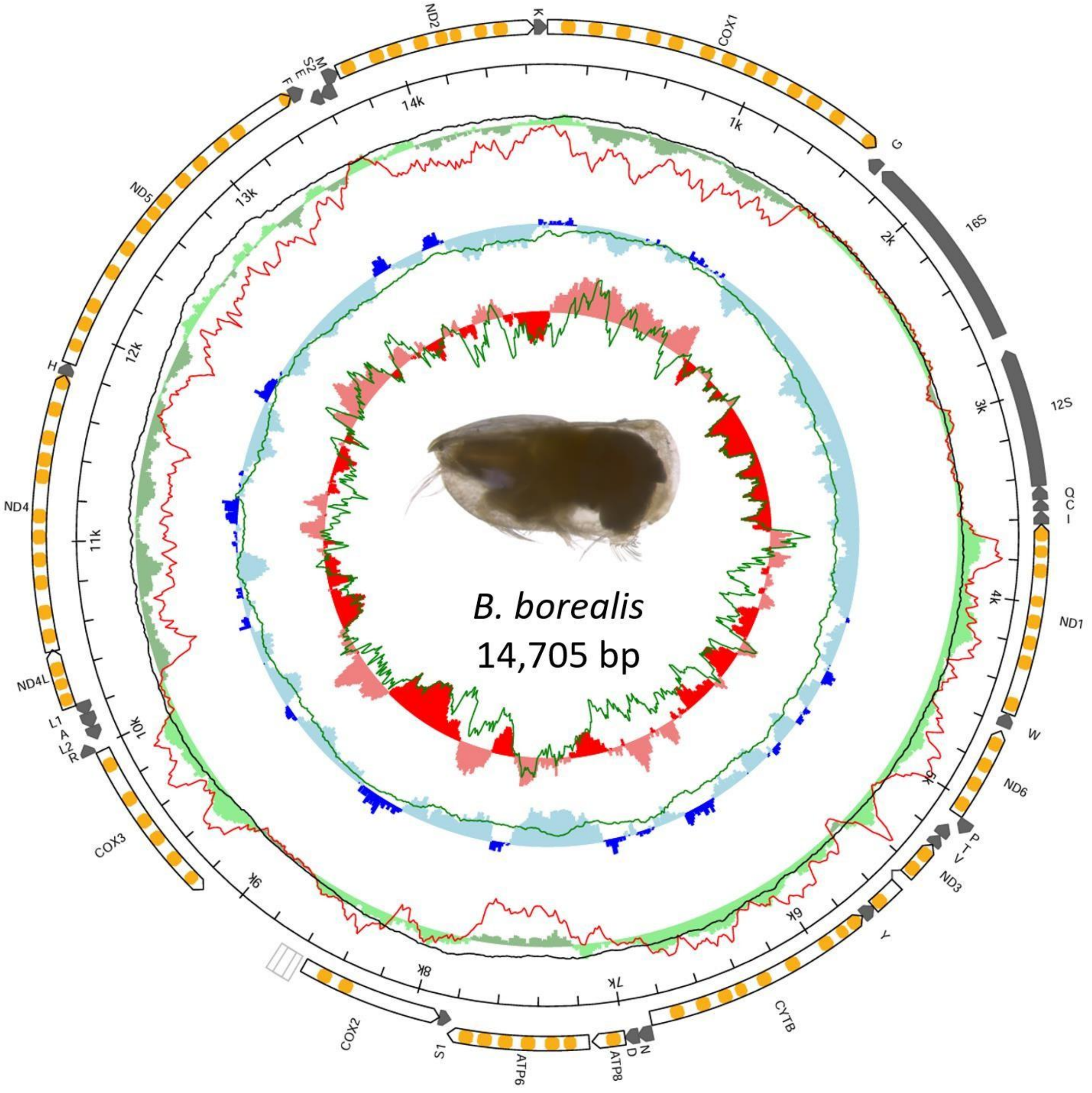

B. borealis
14,705 bp
COX1
G
16S
12S
Q
C
I
ND1
W
ND6
P
T
V
ND3
Y
CYTB
N
D
ATP8
ATP6
S1
COX2
COX3
R
L2
A
L1
ND4L
ND4
H
ND5
F
E
S2
M
ND2
K
1k
2k
3k
4k
5k
6k
7k
8k
9k
10k
11k
12k
13k
14k

1C.

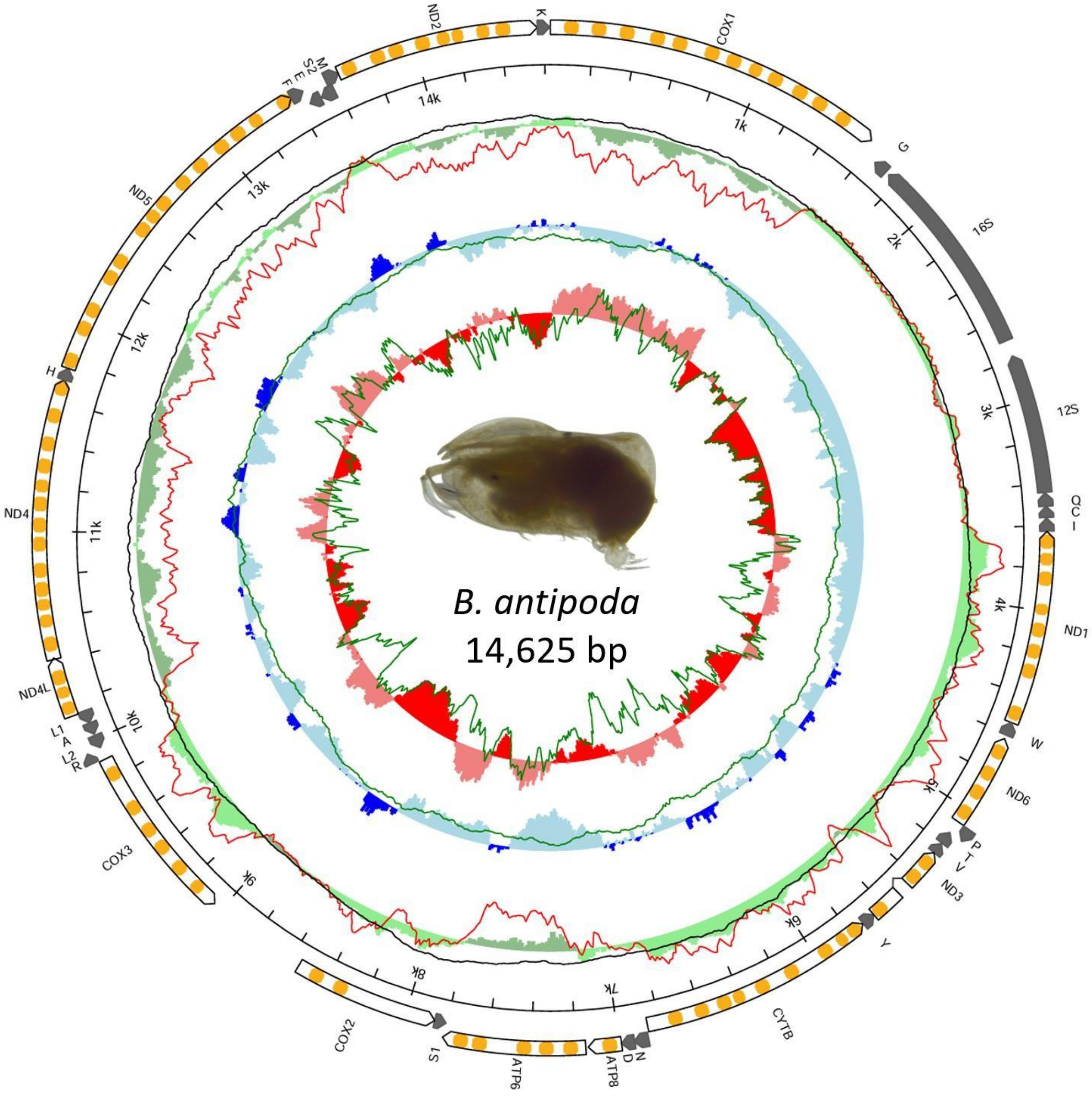
B. antipoda
14,625 bp
COX1
16S
12S
ND1
ND6
ND3
CYTB
ATP8
ATP6
COX2
COX3
ND4L
ND4
ND5
ND2

1D.

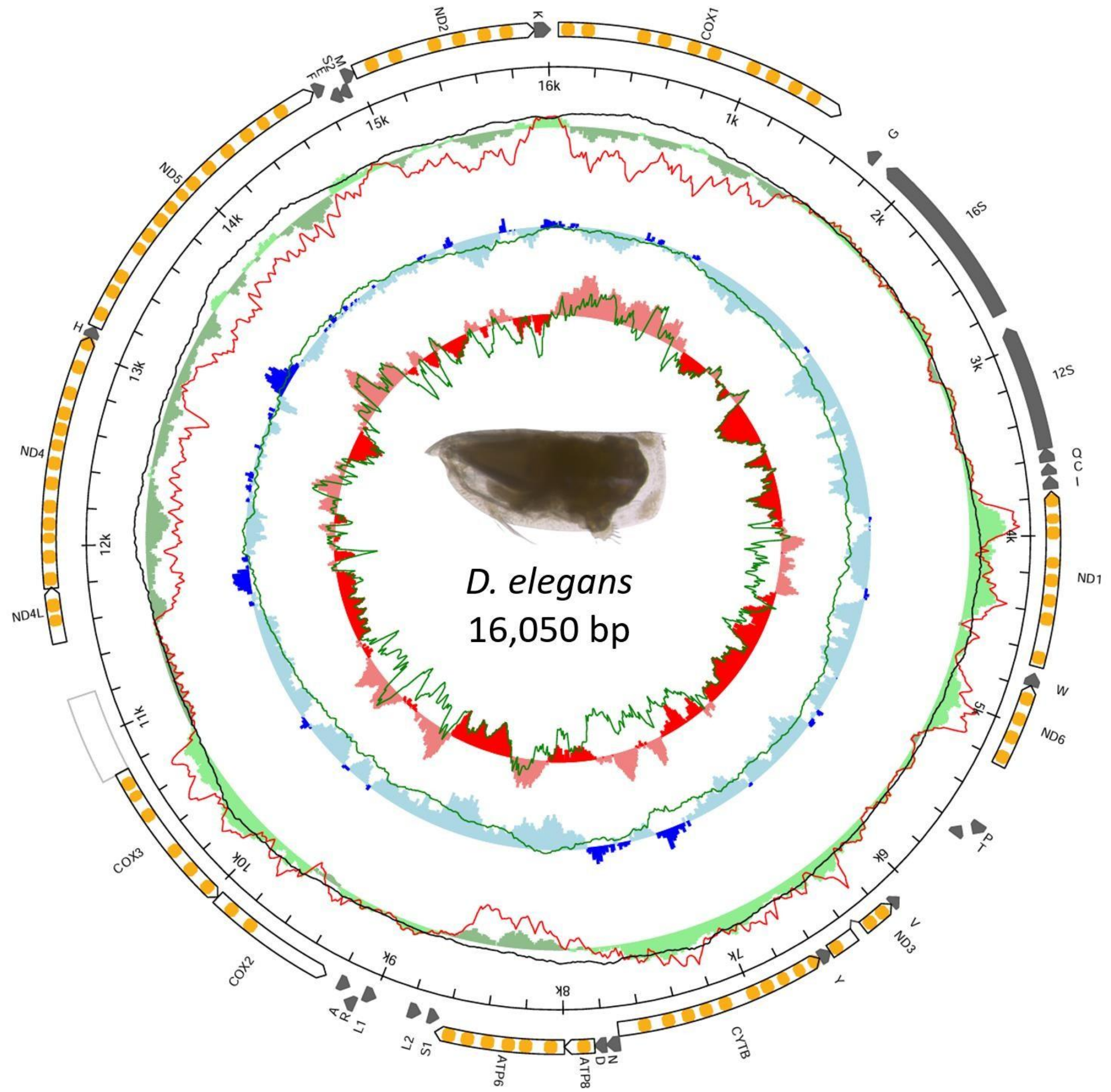

D. elegans
16,050 bp
COX1
G
16S
12S
Q
C
I
ND1
W
ND6
P
T
V
ND3
Y
CYTB
N
D
ATP8
ATP6
S1
L2
L1
R
A
COX2
COX3
ND4L
ND4
H
ND5
F
E
S2
M
ND2
K
16k
1k
2k
3k
4k
5k
6k
7k
8k
9k
10k
11k
12k
13k
14k
15k

1E.

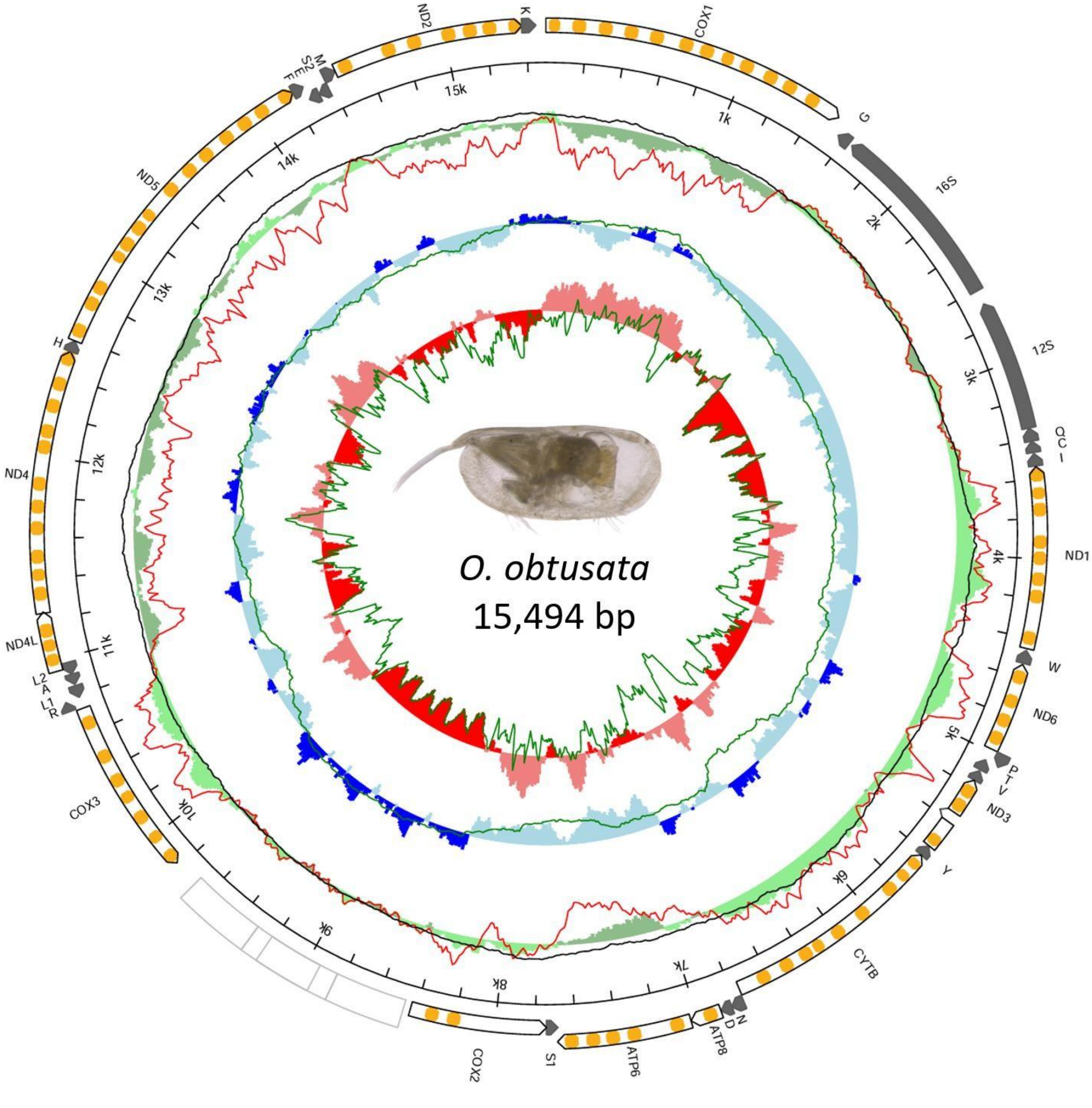

COX1
G
16S
12S
Q
C
I
ND1
W
ND6
P
T
V
ND3
Y
CYTB
N
D
ATP8
ATP6
S1
COX2
COX3
R
L1
A
L2
ND4L
ND4
H
ND5
F
E
S2
M
ND2
K
1k
2k
3k
4k
5k
6k
7k
8k
9k
10k
11k
12k
13k
14k
15k
O. obtusata
15,494 bp

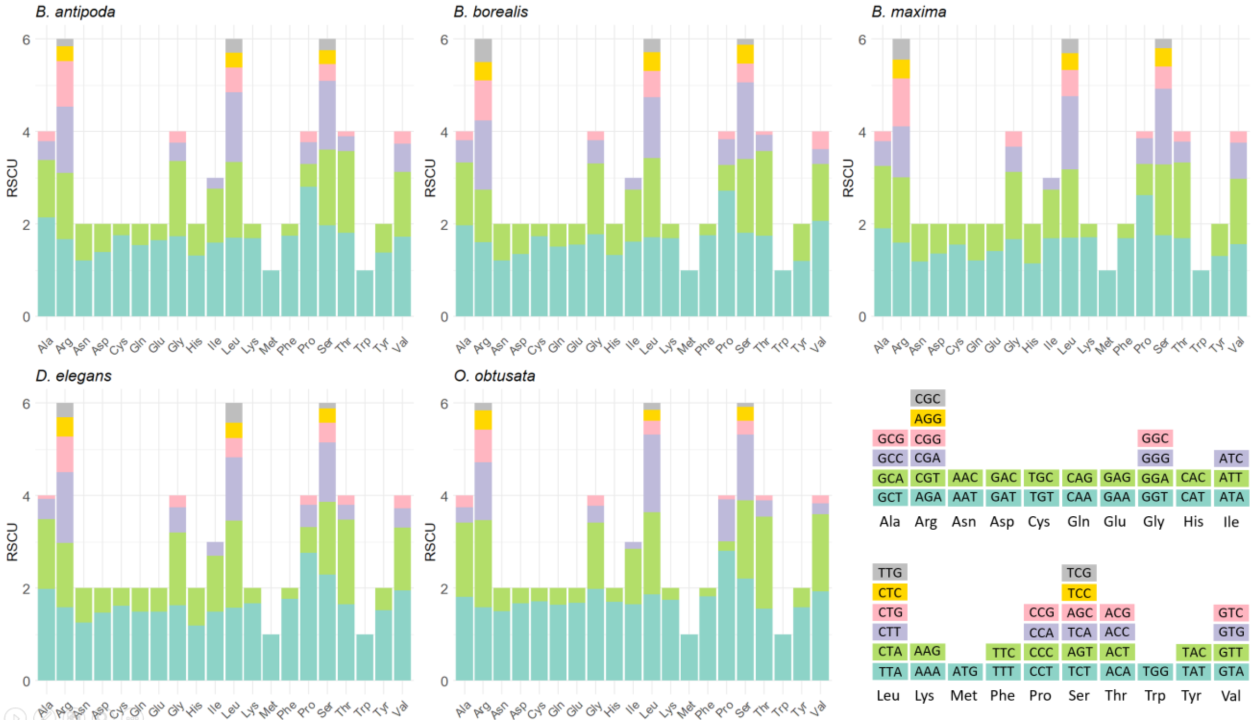

Supplementary Figure 2: RSCU values for amino acids across the five PCGs, measuring how often a particular codon is used relative to other synonymous codons for the same amino acid. The label for the codons that compose each codon family is shown in the boxes on the bottom right corner, and the colors correspond to the colors in the stacked bars. If RSCU = 1, the codon is used equally compared to other synonymous codons, if RSCU > 1, the codon is used more frequently than expected, and if RSCU < 1, the codon is used less frequently than expected.

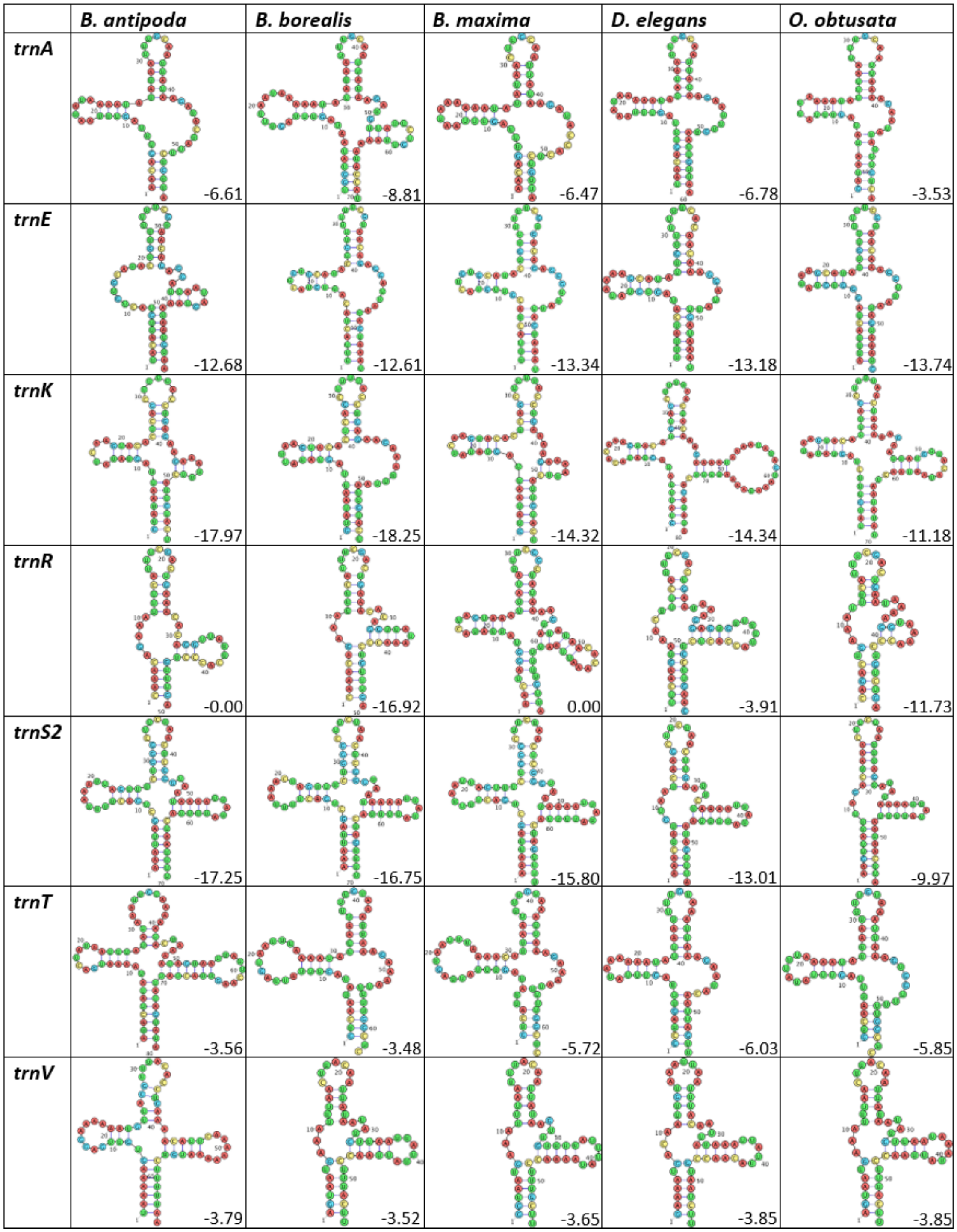

Supplementary Figure 3: Predicted secondary structures of the seven tRNAs that have tRNAs (tRNA-Ala, tRNA-Glu, tRNA-Lys, tRNA-Arg, tRNA-Ser2, tRNA-Thr, and tRNA-Val) that display different structural forms among species, from canonical cloverleafs to loss of specific arms. The values in each box are the ΔG values. The more negative the value, the more thermodynamically stable the secondary structure is.

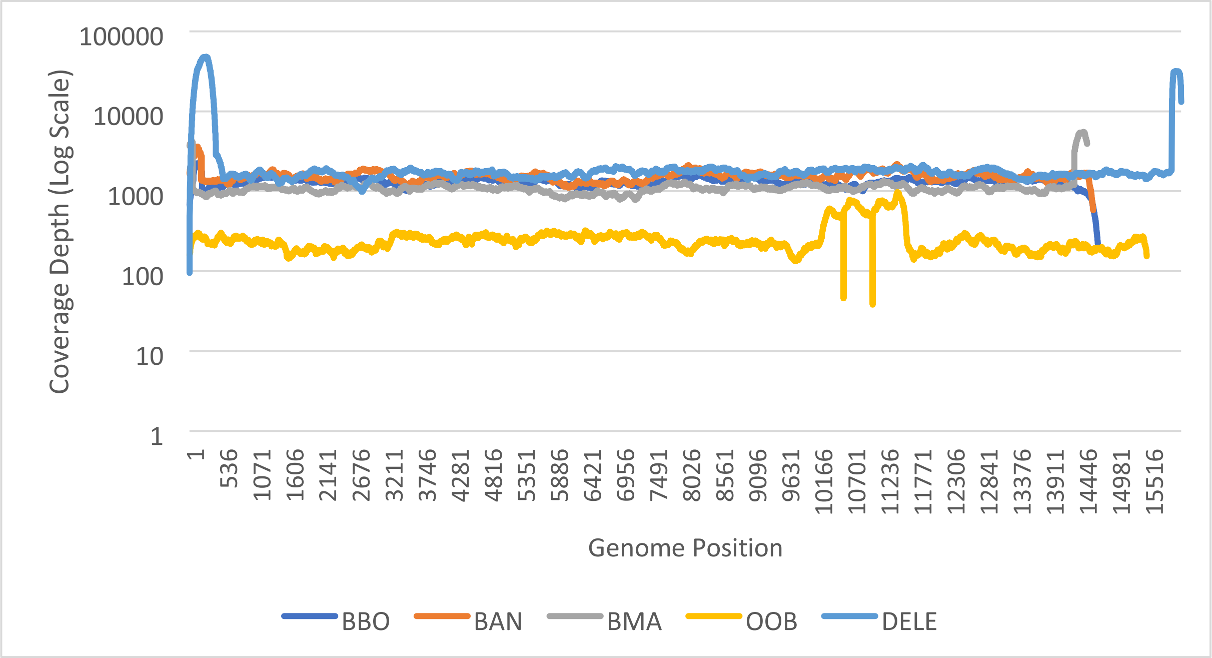

Supplementary Figure 4: Coverage depth plot for the number of sequencing reads aligned to each nucleotide position along the five mitogenomes.

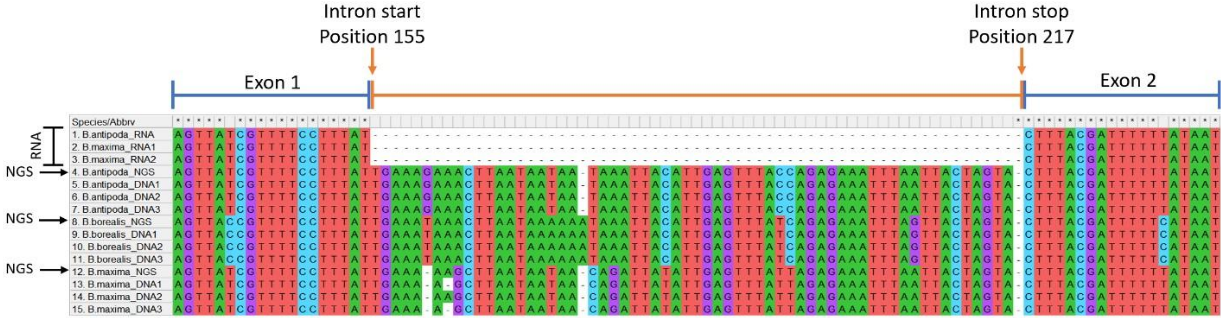

Supplementary Figure 5: *nd3* Sanger sequencing results that show where the intron starts and ends. Nucleotide positions correspond to the genomic *nd3* sequence. Alignment zoomed in to 20 bases before the junction where the exon stops and 20 bases after the junction where the exon resumes. For easier reference, the three individuals used for NGS in this study and the cDNAderived sequences are pointed out.

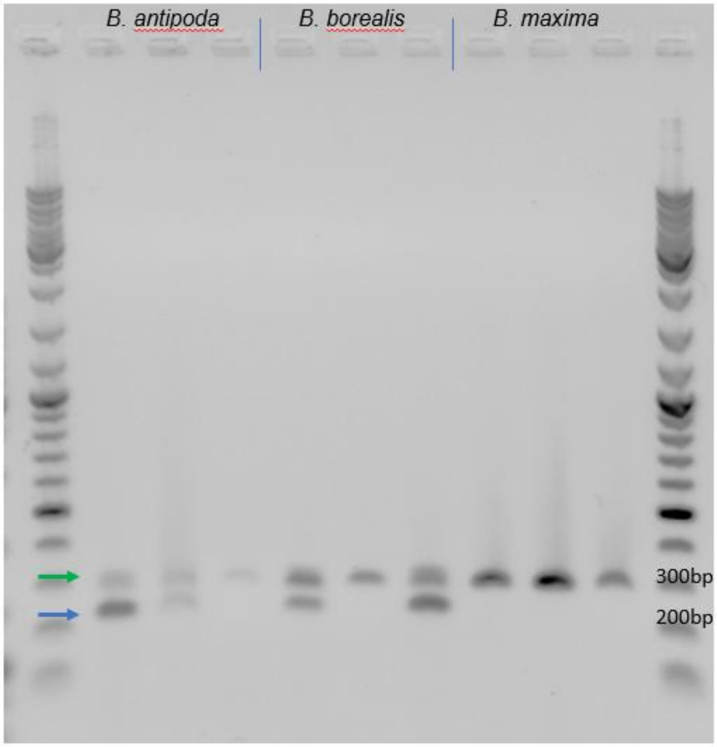

Supplementary Figure 6: Gel electrophoresis results showing the *nd3* RNA to cDNA PCR results. The blue arrow shows the ~250bp product indicating transcripts where the intron was cut out and two parts of the *nd3* transcripts were later are joined together. The green arrow shows the product of expected length, so it is likely a product of DNA contamination or transcript in which intron has not yet been cut out.
